# Recycling Workers: Food Insecurity as a Novel Associated Risk Factor for Toxocariasis

**DOI:** 10.3390/pathogens15090945

**Published:** 2026-09-06

**Authors:** Altina Bruna de Souza Barbosa, Vamilton Alvares Santarém, Cassiana Dahlke Machado, Isabella Braghin Ferreira, Joyce Aparecida da Silva, Susana Angélica Zevallos Lescano, Rogério Giuffrida, Louise Bach Kmetiuk, Alexander Welker Biondo

**Affiliations:** 1 Graduate Colleges of Cell and Molecular Biology, Federal University of Paraná (UFPR), Curitiba 81531-970, PR, Brazil; altinabruna@gmail.com (A.B.d.S.B.); cassianadahlke@gmail.com (C.D.M.); 2 Graduate College in Animal Sciences, University of Western São Paulo (UNOESTE), Presidente Prudente 19067-175, SP, Brazil; vamilton@unoeste.br (V.A.S.); braghinisabella@hotmail.com (I.B.F.); joyceaps@unoeste.edu.br (J.A.d.S.); rgiuffrida@unoeste.br (R.G.); 3 Laboratory of Medical Investigation, Institute of Tropical Medicine of São Paulo, University of São Paulo (USP), Sao Paulo 05403-000, SP, Brazil; suzeles@hotmail.com; 4 Center of Epidemiology, City Secretary of Health, Curitiba 80060-130, PR, Brazil; louisebachk@gmail.com

**Keywords:** larva migrans, tropical neglected diseases, social inequity, *Toxocara* spp., recyclable waste workers

## Abstract

(1) Background: Brazil features one of the largest recycling workforces worldwide, with around 800,000 informal waste pickers, mostly living in urban areas of major cities and dealing with low income and precarious working conditions. This context of vulnerability may lead to a high risk of food insecurity, which may be associated with infectious diseases, particularly toxocariasis, a cosmopolitan neglected parasitic zoonosis with high seroprevalence in tropical countries. (2) Methods: Accordingly, this study assessed *Toxocara* spp. seropositivity and associated risk factors (including food insecurity) in recycling workers of Curitiba, a major Brazilian (fully urban) city currently ranked 8th in population with 1.83 million habitants, 7th in Gross Domestic Product (GDP), and 10th in Human Development Index (HDI) with 0.823 (very high), out of 5569 Brazilian cities. Recycling and non-recycling (used as population control) workers living in the same neighborhood were invited, voluntarily signed consent forms, and had blood serum samples collected and tested by ELISA for detecting anti-*Toxocara* spp. (IgG) antibodies; in addition, an epidemiological questionnaire (socioeconomic characteristics, owning dog/cat, food and hygiene habits) was applied. (3) Results: Overall, 95/177 (53.7%) recycling and 38/100 (38.0%) non-recycling workers tested positive by ELISA, with recycling workers more likely seropositive than non-recycling population (53.7% vs. 38.0%; OR: 1.89; 95% CI: 1.15–3.12%). Food insecurity (OR = 2.01, 95% CI: 1.04–3.91) and low (<1 minimum wage) monthly household income (OR = 3.03, 95% CI: 1.01–9.71) were associated with *Toxocara* spp. seropositivity by multivariate analysis. As expected, cohabitation with both dogs and cats was also significantly associated with seropositivity (OR = 3.75, 95% CI: 1.48–9.75). However, other assessed variables (gender, age, ethnicity, hygienic habits, contact with soil, raw meat ingestion) were not associated with seropositivity. (4) Conclusions: The study herein has filled a gap regarding toxocariasis in vulnerable populations and suggests food insecurity as a socioeconomic indicator for *Toxocara* spp. seropositivity in recycling workers. Furthermore, recycling workers are at higher risk of infection and, as such, need to be prioritized in healthcare programs.

## 1. Introduction

Food insecurity is defined as the violation of the right to regular and permanent access to a sufficient quantity of high-quality food without compromising other essential needs [1]. According to the latest United Nations Bulletin, food insecurity has declined since 2021 but remains a public health concern, with an estimated 28% of the global population living moderately or severely under these conditions [2]. Although it may mostly be found in developing countries, food insecurity is considered a major public health concern in the United States, with approximately 3.8 million (10.5%) households experiencing food insecurity in 2020 [3]. Although zero hunger was included among the 17 Sustainable Development Goals (SDGs) prioritized at the United Nations Summit in 2015 for the 2030 Agenda [4], global malnourishment, hunger, and food insecurity threaten to compromise progress toward not only zero hunger (SDG-2) but all the other SDGs [5].

In the context of vulnerability, some social groups face a higher risk of food insecurity owing to low income and precarious working conditions. Waste pickers or recycling workers (approximately 15 million globally) [6] are considered a highly vulnerable group with low education levels, extreme poverty, job informality, and invisibility, leading to social exclusion and food insecurity [7]. A meta-analysis on food insecurity and the nutritional status of recycling workers in Latin America and Africa revealed that Brazil has the highest food insecurity prevalence among recycling waste collectors [1].

Food insecurity is associated with numerous adverse health outcomes, including chronic disorders such as hypertension, cardiovascular diseases, diabetes, psychiatric disorder such as anxiety and depression [8,9,10,11], and sexually transmitted infections [12]. Although an occupational hazard associated with *Toxocara* spp. exposure was verified in waste pickers in Mexico [13], no study has evaluated the association between *Toxocara* spp. seropositivity and food insecurity in vulnerable populations. In such a scenario, despite its relevance for public health, food insecurity remains to be fully established as a risk factor for zoonotic parasitic infections, particularly toxocariasis in recycling workers.

Toxocariasis is a prevalent but neglected zoonotic disease caused by the larvae of ascarid nematodes (*Toxocara canis* and *Toxocara cati*) in dogs and cats and is mainly transmitted by accidental ingestion of infective eggs present in soil/vegetables or raw meat organs/tissues of paratenic hosts containing larvae (e.g., bovine, chicken) [14]. After eggs are ingested, they hatch and release larvae that penetrate the intestinal wall, reach the liver, migrate throughout the human body, and may cause multiple clinical symptoms, including visceral, ocular, or neurological syndrome [14].

Toxocariasis is one of the most prevalent neglected infections, particularly in populations living in tropical areas and those with social vulnerability [15]. In addition, Brazil has a high *Toxocara* spp. seroprevalence in vulnerable populations, such as 45.9% in homeless individuals [16] and 73.7% in animal hoarders [17]. In total, 229,568 recycling workers are estimated to live in Brazil [6]. However, to date, no study has assessed the risk factors for toxocariasis. Accordingly, this study assessed *Toxocara* spp. seropositivity and associated risk factors (including food insecurity) in recycling workers of Curitiba, a major Brazilian (fully urban) city currently ranked 8th in population with 1.83 million inhabitants, with non-recycling workers living in the same neighborhood used as a comparative population.

## 2. Materials and Methods

### 2.1. Ethical Statement

This study was approved by the Research Human Ethics Committee (CAAE: 68636123.3.0000.0102; report number 6.113.318/2023) of the Federal University of Paraná (UFPR).

### 2.2. Study Area and Population Sampling

This study was conducted in Curitiba (25°25′47″ S, 49°16′19″ W), the capital of Paraná State and a major Brazilian (fully urban) city. Out of 5569 Brazilian cities, Curitiba is currently ranked 8th in population (1.83 million inhabitants), 7th in Gross Domestic Product (GDP), and 10th in Human Development Index (HDI, 0.823—very high). The city is internationally recognized for its sustainability and innovative waste management programs, ranking among the top 20 most sustainable cities worldwide [18]. Curitiba currently has contracts with 49 recycling associations that employ approximately 900–1000 registered recycling workers. A significant concentration of these activities occurs in the Parolin neighborhood, which houses 11 of these associations, with a high density of recycling warehouses and informal recycling workers. Thus, the Parolin neighborhood was selected as the major city area for such economic occupations, and recycling and non-recycling (resident) workers were recruited as participants.

Considering the estimated city population of 1000 recycling workers and the absence of prior local seroepidemiologic data for this specific group, an expected seropositivity rate of 50% was assumed to maximize the statistical power. Applying the Wald method with a 95% confidence level and an absolute precision of ±5%, the minimum required sample size was determined to be 278 participants. The calculation was performed using the epiR package in RStudio v.2025.05.0 (Posit Software, 2026). The study population comprised two closely related groups sharing a common environmental risk profile: recycling workers (*n* = 177 analyzed out of 178 enrolled) and non-recycling (resident) workers of the Parolin neighborhood (*n* = 100). Individuals in the control group were selected from separate households within the same neighborhood. These participants represented community residents engaged in non-recycling activities, as well as homemakers or unemployed individuals. Cohorts shared the same baseline socio-environmental setting, marked by open-air waste accumulation and high companion animal density. Thus, non-recycling controls reflect the baseline community-level environmental exposure, providing a comparative basis for isolating the specific occupational risk associated with direct waste handling. The combined analyzed sample (*n* = 277) was adopted to capture a comprehensive representation of the waste-exposed community, as both groups were subjected to similar ecological pressures and zoonotic transmission pathways for *Toxocara* spp. because of the high density of recycling warehouses and shared urban infrastructure.

### 2.3. Sample Collection

Blood samples were collected after a detailed explanation of the study’s objectives, risks, and benefits to the participants. Venipuncture was performed only after the participants read, understood, and voluntarily signed the Informed Consent Form, ensuring full compliance with the national ethical guidelines. Human peripheral blood samples were obtained via cephalic vein puncture, conducted by certified nurses at the local Primary Health Care Unit. Before blood sampling, the venipuncture site was thoroughly cleaned and disinfected to minimize the risk of contamination. The blood samples were placed into sterile tubes containing a serum-separating gel without anticoagulant and centrifuged at 5000 RPM for 5 min. The serum was stored at −80 °C until processing.

### 2.4. Epidemiological Data

Potential risk factors associated with anti-*Toxocara* spp. antibody seroprevalence were tested using a comprehensive and standardized epidemiological questionnaire that was administered to all participants. The questionnaire was designed to assess demographic and socioeconomic characteristics as well as occupational habits. Environmental and behavioral factors were also included, such as the frequency of contact with domestic animals (dogs and cats) and soil, hand hygiene practices, and consumption of unwashed vegetables.

The risk of household food insecurity was assessed based on the Food Insecurity Screening Tool (TRIA), a validated instrument specifically developed to identify the risk of food insecurity rather than providing a graded clinical diagnosis, as performed by the Brazilian Food Insecurity Scale (EBIA) [19]. The screening consisted of two objective questions (yes/no): (1) In the last three months, did food supplies run out before the family had money to buy more? and (2) In the last three months, did you have to eat only certain types of food because the money ran out? By aligning this assessment with the biological persistence of anti-*Toxocara* spp. IgG antibodies, which indicate past or chronic exposure, the recall period was adapted from the validated 3-month window to assess cumulative lifetime exposure, functioning as an exploratory proxy for lifetime socio-environmental vulnerability rather than a standard clinical scale. Following the protocol, participants were classified as ‘positive’ for the risk of food insecurity if they gave an affirmative answer (yes) to at least one of the two questions.

### 2.5. Serology

An enzyme-linked immunosorbent assay (ELISA) was performed using 96-well polystyrene microplates (Corning, Costar, Ref. 3590, New York, NY, USA) coated with *Toxocara* excretion-secretion antigens (TES) at a concentration of 1.9 μg/μL per well. As previously stated, the ELISA used *Toxocara* excretory-secretory antigen (TES) obtained from *T. canis* larva. Preincubation of serum with adult *Ascaris suum* extract was adopted to minimize cross-reactive antibodies from other ascarid parasites [20]. TES antigens were harvested from L3 larvae maintained in Roswell Park Memorial Institute (RPMI) culture medium. Coating was performed in a 0.06 M carbonate-bicarbonate buffer (pH 9.6), in initial incubation at 37 °C for 2 h, followed by overnight incubation at 4 °C. The plates were rinsed three times with phosphate-buffered saline containing Tween 20 (PBS-T) and subsequently blocked with PBS-T supplemented with 5% skimmed milk (Molico^®^, Nestlé, Vevey, Switzerland) for 1 h at 37 °C.

To reduce potential cross-reactivity, serum samples were pre-adsorbed with *Ascaris suum* somatic antigens (25 μg/mL) diluted in the blocking buffer. Thereafter, the serum samples were diluted 1:200 in the same solution, pre-incubated at 37 °C for 30 min, and transferred in duplicate to sensitized plates. The plates were then incubated for 1 h at 37 °C. After a triple wash cycle, a peroxidase-conjugated goat anti-human IgG antibody (Sigma A6029, St. Louis, MO, USA) was incorporated at a 1:5000 dilution and incubated for 60 min at 37 °C. The wells were washed three times for 5 min each. Colorimetric development was initiated by adding 3,3′,5,5′-tetramethylbenzidine (TMB) substrate (Thermo Fisher Scientific, Waltham, MA, USA) to the enzymatic reaction and subsequently stopped using 2 N sulfuric acid. All assay plates contained verified positive and negative control sera provided by the biorepository of the Institute of Tropical Medicine at the University of São Paulo.

The optical density (OD) was measured at 450 nm, and the diagnostic threshold (cutoff) was established as the mean absorbance of 90 negative controls plus three standard deviations. The final antibody levels were determined as reactivity indices (RI), which were calculated by dividing the OD of each test sample by the threshold value. Serum samples with an RI greater than 1 were considered positive [21].

### 2.6. Statistical Analysis

Bivariate analysis followed by a logistic regression model (multivariate) was used to evaluate the seropositivity of *Toxocara* spp. First, the epidemiological and serological information was entered into an Excel spreadsheet. A bivariate model using Pearson’s chi-squared test was used to verify the association between categorical variables and seropositivity. Odds ratios (OR) and 95% confidence intervals (95% CI) were calculated based on raw data. Variables resulting in a *p*-value less than 0.2 in the bivariate model were considered eligible for inclusion in a multivariate logistic regression model. A backward stepwise method adopting Akaike’s information criterion (AIC) was employed to select predictors of seropositivity. The final model coefficients were used to calculate the OR with 95% confidence intervals (CI) using the Wald method. The predictive capacity of the final model was verified by calculating the area under the ROC curve (and the respective CIs) using the DeLong method, and collinearity was verified by calculating the variance inflation factor (VIF). All analyses were performed in R version 4.6.1 [22] using the Blorr and CAR packages [23].

## 3. Results

### 3.1. Population Characteristics

The outcome population consisted of 277 participants, including 177 recycling workers and 100 non-recycling workers (population contrast). From a socio-environmental perspective, both study groups reside within the same geographic territory, an area characterized by a high concentration of recycling facilities and shared socioeconomic status. A common characteristic observed in all the recycling warehouses surveyed was the on-site presence of at least one domestic dog or cat that was intentionally kept by recycling workers as a rodent control strategy. Thus, ubiquitous exposure to companion animals within the occupational milieu was reported by all recycling workers, regardless of non-occupational pet ownership.

Although female participants predominated in both study groups (104/177 (58.4%) recycling and 76/100 (76.0%) non-recycling workers), a significant association was observed between sex and occupation (*p* = 0.004). Specifically, male representation was significantly higher among recycling workers (41.6%) than among non-recycling workers (24.0%).

The recycling worker group had lower educational levels, with 83.6% reporting interrupting their studies in elementary school. In contrast, the non-recycling worker group (living in the same neighborhood) exhibited a heterogeneous profile with a higher distribution of advanced qualification tiers, with approximately 58% reporting completion of at least high school. Consequently, a significant association was found between education level and occupation, with individuals without a high school degree showing 7.047-fold higher odds of working in recycling when compared to those with higher educational levels (OR = 7.047; 95% CI: 4.02–12.37; *p* < 0.001) (Table 1). No statistically significant association was found for non-recycling workers (Supplementary Table S1).

### 3.2. Seroprevalence and Associated Risk Factors for Seropositivity

One sample was excluded because of insufficient serum volume required to perform the ELISA in triplicate, which was maintained to guarantee technical reproducibility and precision. Thus, although 178 recycling workers were initially enrolled, only 177 were included in the final serological analysis.

The overall seropositivity of anti-*Toxocara* spp. IgG among the studied recycling workers of 53.7% (95/177; 95% CI: 46.3–60.9) was significantly higher (OR: 11.89; 95% CI: 1.15–3.12; *p* = 0.017) than the 38.0% (38/100; 95% CI: 29.1–47.8) verified in non-recycling workers. Considering the recycling workers group, multivariate analysis revealed that low socioeconomic status was an associated risk factor for anti-*Toxocara* seropositivity. Individuals with household income of less than one minimum wage faced a three-fold increase in likelihood of seropositivity (OR = 3.03, 95% CI: 1.01–9.71, *p* = 0.048). Furthermore, food insecurity was found as an independent associated risk factor, increasing the odds of seropositivity two-fold (OR = 2.01, 95% CI: 1.04–3.91, *p* = 0.037).

Among transmission routes, contact with soil was reported by 32/95 (33.7%) seropositive and 21/82 (25.6%) seronegative workers, showing no statistically significant association with anti-*Toxocara* seropositivity (OR = 1.47, 95% CI: 0.77–2.86, *p* = 0.315). Likewise, raw meat consumption was reported by 46/95 (48.4%) seropositive and 39/82 (48.1%) seronegative workers, with no significant correlation with seropositivity in the descriptive univariate analysis (OR = 1.01, 95% CI: 0.56–1.84, *p* = 1.000).

The multivariate regression model evaluating domestic characteristics showed that cohabitation with both dogs and cats was associated with seropositivity, increasing the odds of anti-*Toxocara* seropositivity by 2.75-fold (OR = 3.75, 95% CI: 1.48–9.75, *p* = 0.005). In contrast, single-species ownership showed no independent statistically significant association with seropositivity, whether in individuals owning only dogs (*p* = 0.311) or cats (*p* = 0.279) (Table 2).

The logistic regression model demonstrated poor discriminative ability, with an area under the curve (AUC) of 0.665 (95% CI: 0.586–0.744), indicating that the model performed more regularly than chance at distinguishing between outcome categories. No significant VIF was detected for the variables retained in the final model (1.020 for having a dog/cat or both categories and 1.050 for family income and food insecurity).

## 4. Discussion

This study is the first serosurvey to address the risk factors associated with *Toxocara* spp. seropositivity in recycling workers (recyclable waste collectors) in Brazil and worldwide. The seropositivity observed herein in recycling workers (95/177; 53.7%) was around 10% higher than that observed in residents of a nearby low-income neighborhood (58/132; 43.9%) in the same metropolitan area of Curitiba [24] and 25.7% higher than the general population of Brazil (28%; 95% CI: 22.0–34.0%), according to a meta-analysis estimate [25]. In addition, seropositivity was 40.7% higher than that observed in waste pickers (12/90; 13%) in Mexico [13]. In Brazil, an 8.8% (95% CI: 5.5–12) estimated prevalence of *T. canis* infection in dogs has been estimated according to a global meta-analysis [26,27]. Another global meta-analysis revealed that dog contact represents a risk factor for *Toxocara* spp. infection in individuals under 18 years old [28]. Herein, no attempt was made to analyze fecal or serological samples of recyclers’ dogs to evaluate the infection or exposure to *Toxocara canis*, respectively, an issue that limits the ability to infer the risk of infection of participants by dog contact.

Most of the recycling workers (101/177; 57.1%) were considered to live under food insecurity conditions, presenting higher seropositivity rate when compared with non-recycling workers (38/100; 38%; OR = 1.89; 95% CI: 1.15–3.12), corroborating a serosurvey in Mexico [13]. These findings indicate *Toxocara* spp. as another occupational risk for recycling workers, in addition to other occupation-related health problems due to infectious diseases [6,29].

In addition to the high *Toxocara* spp. seroprevalence, the present study found food insecurity to be a novel associated risk factor for seropositivity among recycling workers. Logistic regression revealed a higher chance (OR = 2.3) of recycling workers under food-insecure conditions having a positive ELISA test when compared with non-recycling participants. In addition, low familial income was also revealed as an associated risk factor for seropositivity according to logistic regression analysis. Food insecurity and poverty are considered two mutually reinforcing conditions, indicating that poor households are significantly more likely to be affected by food insecurity [30]. Food insecurity is globally recognized as a key socioeconomic determinant of a wide spectrum of health outcomes [31,32]. Thus, our results indicate that food insecurity should be included in future serosurveys as a potential associated risk factor for toxocariasis, along with family income and educational levels [33,34,35].

As one of the most common times to be infected by *Toxocara* larvae is as a child, food insecurity may have occurred then along with infection, with a relatively higher rate of infection when compared to those children with food security. However, as food insecurity herein was statistically significant when comparing the groups as a whole, further studies should increase child samplings and fully establish whether food insecurity impacts on *Toxocara* infection during childhood. *Toxocara* larvae can survive in human and animal tissues for years, continually releasing excretory-secretory antigens that stimulate the host to maintain specific antibody production [36]. Thus, although standard IgG antibodies have a half-life in human plasma of around three weeks, due to ongoing antigenic stimulation from living larvae lodged in tissues, antibodies are continuously refilled by the host’s immune system and may remain elevated for months or years after infection, declining very slowly only when the parasite dies or stops releasing antigens.

Other socioeconomic population characteristics evaluated in the present study (sex, ethnicity, and age) were not associated with *Toxocara* spp. seropositivity. As the recycling worker group was composed mostly of adults, no influence of age was observed on seropositivity, corroborating previous studies on adult individuals [33,37,38]. Across all surveyed facilities, workers handled a consistent mix of dry household recyclables—predominantly paper, cardboard, plastics, glass, and metals—without facility-specific specialization in materials that could disproportionately drive the exposure risk. Nonetheless, higher seropositivity in adult female housewives (when compared to the male group) was reported in South Iran, which was associated with contaminated vegetables and contact with pets [38]. However, as the recycling workers evaluated in this study worked strictly in urban settings, women (and men) had low or no exposure to agricultural activity. Therefore, the lack of statistical significance in sex, age, and ethnicity suggests that exposure risk was widespread and heavily driven by shared socio-environmental conditions rather than individual demographic traits.

Concomitant ownership of dogs and cats was revealed to be an associated risk factor for toxocariasis, similar to findings in the Brazilian elderly population (over 50 years old) showing that dog and cat ownership (but not cat or dog ownership) increased the odds (OR = 3.1) of toxocariasis [33]. However, these findings contrast with those of a vulnerable population living in the metropolitan area of Curitiba, where the presence of dogs, cats, or both was not associated with seropositivity. According to a global meta-analysis, owning cats or dogs represents a risk factor for people under 18 but not for adults [28]. However, studies based on Latin America and the Caribbean revealed the presence of dogs at home as a risk for the general population [25]. Although such individuals may be exposed to *Toxocara* spp. and other zoonoses due to their close contact with pets, who are mostly not provided with veterinary care, unnourished, and lacking deworming/vaccination [28], the positive mental (loneliness and stress reduction) and social (interaction and sense of community) benefits promoted by pets have been undeniable [39].

Human toxocariasis is widely recognized as a soil-transmitted zoonosis (via the ingestion of embryonated eggs) and food-borne infection (via the consumption of encysted larvae in paratenic hosts) [40,41]. Therefore, variables such as direct contact with soil and ingestion of raw meat have traditionally been considered primary risk factors. However, in the present study, neither of these two variables was statistically associated with seropositivity. This lack of significance suggests that within this specific population of recycling workers, occupational exposure and broader socio-environmental vulnerabilities, rather than isolated dietary or individual behavioral habits, may play a more significant role in driving the high observed seroprevalence.

Limited access to formal education is the structural basis of social vulnerability. The association between low educational level and recycling occupation showed that a lack of high school increased the likelihood of working in recycling by 7.03-fold. These findings may indicate that recycling activities serve as a survival strategy and income source for individuals without educational requirements for the formal labor market. From a public health perspective, recycling workers’ restricted access to formal education raises a critical concern, as such a deficit may lead to low healthcare education and low awareness of preventive information.

### Study Limitations

The present study has several limitations. First, as the associated risk factor assessment was based on self-declared information from both recycling and non-recycling workers, the possibility of biased responses cannot be ruled out. In addition, higher male (than female) refusal to participate, for example, due to apprehension about the blood samplings (fear of needles), may have underestimated or biased the sex comparison. However, adherence was higher among male recycling workers who recognized the study’s potential to support future public health policies for their category. By contrast, non-recycling residents lacked this specific occupational motivation, leading to a higher refusal rate and contributing to the observed gender disparity. Additionally, the ELISA used in this study did not differentiate between recent and chronic infections in the tested individuals. However, to minimize cross-reactivity and reduce false-positive results with *Ascaris lumbricoides* antibodies, pre-adsorption with *A. suum* was used—a methodology adopted in other studies performed by our research group [42,43,44] in a reference laboratory for toxocariasis diagnosis in Brazil.

Another methodological limitation was the adaptation of the TRIA. This adaptation introduces a potential recall bias, as participants may have underestimated the historical periods of food hardship over their lifespan, creating a risk of exposure misclassification. Although this modification was necessary to capture cumulative life-course vulnerability in a cross-sectional serological design, caution is warranted when comparing these prevalence figures with studies utilizing standardized short-term food insecurity scales.

## 5. Conclusions

In summary, this study provides new insights into toxocariasis in vulnerable populations, those with occupational exposure, and recycling workers, revealing a novel association between seropositivity and food insecurity, along with low familial income. Future studies should be conducted to fully establish these associations with recycling workers and other underprivileged socioeconomic populations in Brazil and worldwide to provide a more comprehensive assessment of food insecurity as an independent risk factor for toxocariasis.

## Figures and Tables

**Table 1:** Association between educational level and occupational group of recycling and non-recycling workers.

Worker Educational Level	Recycling	Non-Recycling	OR	IC 95%	*p*-Value
High school or higher	29 (16.4%)	58 (58.0%)	1.0	-	-
Up to elementary school	148 (83.6%)	42 (42.0%)	7.047	4.02–12.37	<0.001 *

* Note: Fisher’s exact test exact *p*-value = 0.00000000000136.

**Table 2:** Seropositivity and associated risk factors between anti-IgG against *Toxocara* spp. in recycling workers in southern Brazil.

	Positive *n* (%)	Negative *n* (%)	Univariate Analysis		Multivariate Analysis	
Characteristics	95 (53.7)	82 (46.3)	OR (95% CI)	*p*	OR (95% CI)	*p*
Gender				0.947	NC	
Female	56 (58.9)	47 (57.3)	1 [Reference]			
Male	39 (41.1)	35 (42.7)	0.94 (0.51–1.71)			
Age * (interquartile)				0.371	NC	
17–27	19 (20.0)	25 (30.9)	1 [Reference]			
28–38	27 (28.4)	17 (21.0)	2.07 (0.88–4.94)			
39–46	24 (25.3)	19 (23.5)	1.65 (0.71–3.92)			
47–72	25 (26.3)	20 (24.7)	1.63 (0.70–3.84)			
Ethnicity				0.869	NC	
White	36 (37.9)	33 (40.2)	1 [Reference]			
Non-white	59 (62.1)	49 (59.8)	1.1 (0.60–2.03)			
Familiar income (minimum wage)				0.029		
2.2 or more	6 (6.3)	16 (19.5)	1 [Reference]		1 [Reference value]	
One up to 2.1	43 (45.3)	33 (40.2)	3.39 (1.23–10.51)		2.82 (0.93–9.09)	0.067
Less than one	46 (48.4)	33 (40.2)	3.62 (1.32–11.20)		3.03 (1.01–9.71)	0.048
Food Insecurity				0.013		
No	29 (30.5)	41 (50.0)	1 [Reference]		1 [Reference value]	-
Yes	66 (69.5)	41 (50.0)	2.26 (1.23–4.23)		2.01 (1.04–3.91)	0.037
Handwashing before meals				0.273	NC	
No	6 (6.3)	10 (12.2)	1 [Reference]			
Yes	89 (93.7)	72 (87.8)	2.03 (0.71–6.35)			
Raw meat intake *				1	NC	
No	49 (51.6)	42 (51.9)	1 [Reference]			
Yes	46 (48.4)	39 (48.1)	1.01 (0.56–1.84)			
Contact with soil				0.315	NC	
No	63 (66.3)	61 (74.4)	1 [Reference]			
Yes	32 (33.7)	21 (25.6)	1.47 (0.77–2.86)			
Biting nails *				1	NC	
No	71 (74.7)	60 (74.1)	1 [Reference]			
Yes	24 (25.3)	21 (25.9)	0.97 (0.49–1.92)			
Raising pets						
No	18 (18.9)	26 (31.7)	1 [Reference]	0.035	1 [Reference value]	-
Dog	40 (42.1)	39 (47.6)	1.47 (0.70–3.15)		1.49 (0.69–3.26)	0.311
Dog and cat	30 (31.6)	12 (14.6)	3.53 (1.45–9.01)		3.75 (1.48–9.75)	0.005
Cat	7 (7.4)	5 (6.1)	1.98 (0.53–7.90)		2.1 (0.55–8.57)	0.279
Rinsing vegetables before meal				0.315	NC	
None	5 (5.3)	9 (11.0)	1 [Reference]			
Running water	59 (62.1)	51 (62.2)	2.04 (0.65–7.21)			
Chemical product	31 (32.6)	22 (26.8)	2.47 (0.73–9.27)			

* Missing data: one missing data for each tested characteristic; NC: not calculated. No fit for multivariate analysis (*p*-value higher than 0.2).

## Data Availability

The raw data supporting the conclusions of this article will be made available by the authors on request.

## Supplementary Materials

The following supporting information can be downloaded at: https://www.mdpi.com/article/10.3390/pathogens15090945/s1, Supplementary Table S1: Seropositivity and associated risk factors between anti-IgG against *Toxocara* spp. in non-recycling workers in southern Brazil.

## References

[1] Pedra L.E.Q., de Oliveira Pinto V.S., Ferreira A.A., Guimarães N.S. (2025). Food insecurity and nutritional status of Latin American and African waste pickers: A systematic review and meta-analysis. Syst. Rev..

[2] SDG Indicators. https://unstats.un.org/sdgs/report/2025/Goal-02/.

[3] Pasha V.C., Gerchow L., Lyndon A., Clark-Cutaia M., Wright F. (2024). Understanding Food Insecurity as a Determinant of Health in Pregnancy Within the United States: An Integrative Review. Health Equity.

[4] THE 17 GOALS|Sustainable Development. https://sdgs.un.org/goals.

[5] Moramarco S., Buonomo E., Andreoli A., Palombi L. (2025). Tackling Global Malnutrition and Hunger in the Final Push Toward the 2030 Agenda. Nutrients.

[6] Zolnikov T.R., da Silva R.C., Tuesta A.A., Marques C.P., Cruvinel V.R.N. (2018). Ineffective waste site closures in Brazil: A systematic review on continuing health conditions and occupational hazards of waste collectors. Waste Manag..

[7] Souza-Silva G., Mol M.P.G. (2021). Hepatitis B or C prevalence in waste pickers from South America: A systematic review. J. Public Health.

[8] Militao E.M.A., Salvador E.M., Uthman O.A., Vinberg S., Macassa G. (2022). Food Insecurity and Health Outcomes Other than Malnutrition in Southern Africa: A Descriptive Systematic Review. Int. J. Environ. Res. Public Health.

[9] Parekh T., Xue H., Cheskin L.J., Cuellar A.E. (2022). Food insecurity and housing instability as determinants of cardiovascular health outcomes: A systematic review. Nutr. Metab. Cardiovasc. Dis..

[10] Osei-Owusu C., Dhillon S., Luginaah I. (2024). The impact of food insecurity on mental health among older adults residing in low- and middle-income countries: A systematic review. PLoS ONE.

[11] Radtke M.D., Steinberg F.M., Scherr R.E. (2024). Methods for Assessing Health Outcomes Associated with Food Insecurity in the United States College Student Population: A Narrative Review. Adv. Nutr..

[12] Balistreri K.S., Lyons H.A. (2026). Food Insecurity and Sexually Transmitted Infections in the United States. Sex. Transm. Dis..

[13] Alvarado-Esquivel C. (2013). Toxocariasis in waste pickers: A case control seroprevalence study. PLoS ONE.

[14] Chen J., Liu Q., Liu G.-H., Zheng W.-B., Hong S.-J., Sugiyama H., Zhu X.-Q., Elsheikha H.M. (2018). Toxocariasis: A silent threat with a progressive public health impact. Infect. Dis. Poverty.

[15] Lopez-Alamillo S., Padyala P., Carey M., Duffey M.M., Weatherhead J.E. (2025). Human toxocariasis. Clin. Microbiol. Rev..

[16] Santarém V.A., do Couto A.C., Lescano S.Z., Roldán W.H., Delai R.R., Giuffrida R., Kmetiuk L.B., Biondo A.W., Dangoudoubiyam S., Dos Santos A.P. (2022). Serosurvey of anti-*Toxocara canis* antibodies in people experiencing homelessness and shelter workers from São Paulo, Brazil. Parasit. Vectors.

[17] Santarém V.A., Kmetiuk L.B., Ferreira I.B., Lescano S.A.Z., de Souza Filho R.T., da Cunha G.R., Morikawa V.M., Dangoudoubiyam S., Pires Dos Santos A., Biondo A.W. (2024). Seropositivity for *Toxocara* spp. in Individuals with Animal Hoarding Disorder in Southern Brazil: An Alarm for Public Health. Acta Parasitol..

[18] Prince C.S. Sustainable Cities Index 2022. Corporate Knights. https://corporateknights.com/sustainable-cities/.

[19] Poblacion A., Segall-Corrêa A.M., Cook J., Taddei J.A.d.A.C. (2021). Validity of a 2-item screening tool to identify families at risk for food insecurity in Brazil. Cad. Saúde Pública.

[20] Nguyen Y.T.H., Hayata Y., Sonoda S., Nonaka N., Maruyama H., Yoshida A. (2020). Establishment of a serodiagnosis system for the detection of *Toxocara* spp. and *Ascaris suum* infection in chickens. Parasitol. Int..

[21] Dziemian E., Zarnowska H., Kołodziej-Sobocińska M., Machnicka B. (2008). Determination of the relative avidity of the specific IgG antibodies in human toxocariasis. Parasite Immunol..

[22] R: The R Project for Statistical Computing. https://www.r-project.org/.

[23] Hebbali A. blorr: Tools for Developing Binary Logistic Regression Models 2024. https://blorr.rsquaredacademy.com/.

[24] Sohn-Hausner N., Correa R.G., Zevallos Lescano S.A., Brime P.S., Giuffrida R., Kmetiuk L.B., Santarém V.A., Biondo A.W. (2026). Socioeconomic vulnerability associated with toxocariasis exposure in southern Brazil: A One Health approach. Front. Public Health.

[25] Ulloque-Badaracco J.R., Hernandez-Bustamante E.A., Alarcón-Braga E.A., Huayta-Cortez M., Carballo-Tello X.L., Seminario-Amez R.A., Rodríguez-Torres A., Casas-Patiño D., Herrera-Añazco P., Benites-Zapata V.A. (2023). Seroprevalence of human toxocariasis in Latin America and the Caribbean: A systematic review and meta-analysis. Front. Public. Health.

[26] Bonilla-Aldana D.K., Morales-Garcia L.V., Ulloque Badaracco J.R., Mosquera-Rojas M.D., Alarcón-Braga E.A., Hernandez-Bustamante E.A., Al-Kassab-Córdova A., Benites-Zapata V.A., Rodriguez-Morales A.J., Delgado O. (2023). Prevalence of *Toxocara* eggs in Latin American parks: A systematic review and meta-analysis. Infez. Med..

[27] Rostami A., Riahi S.M., Hofmann A., Ma G., Wang T., Behniafar H., Taghipour A., Fakhri Y., Spotin A., Chang B.C.H. (2020). Global prevalence of *Toxocara* infection in dogs. Adv. Parasitol..

[28] Merigueti Y.F.F.B., Giuffrida R., da Silva R.C., Kmetiuk L.B., Santos A.P.D., Biondo A.W., Santarém V.A. (2022). Dog and Cat Contact as Risk Factor for Human Toxocariasis: Systematic Review and Meta-Analysis. Front. Public Health.

[29] Zolnikov T.R., Ramirez-Ortiz D., Moraes H., Cruvinel V.R.N., Dominguez A., Galato D. (2019). Continued Medical Waste Exposure of Recyclable Collectors Despite Dumpsite Closures in Brazil. J. Health Pollut..

[30] Understanding Poverty and Food Insecurity at the Household Level|Agrifood Economics|Food and Agriculture Organization of the United Nations. https://www.fao.org/agrifood-economics/publications/detail/en/c/1626429/.

[31] Laurentino J.S.L., Brito R.C.d.S., Oliveira-Silva R.T.d., Soares A., Pereira T.d.C., Lima E.M.d., Santos A.B.M.V.D., Palmeira P.d.A. (2024). Association between food insecurity and chronic noncommunicable diseases in Brazil: A systematic review. Rev. Bras. Epidemiol..

[32] Dlamini S.N., Mtintsilana A., Craig A., Mapanga W., Norris S.A. (2024). Food insecurity and coping strategies associate with higher risk of anxiety and depression among South African households with children. Public Health Nutr..

[33] da Silva Rapchan G.G., Ferreira I.B., Lima V.D.S.V., Lescano S.A.Z., Monteiro G.R., Santos G.C.D., Esteves L.S.F., Figueiredo F.B., Kmetiuk L.B., Biondo A.W. (2025). Toxocariasis as an Elderly Zoonosis: Seroprevalence, Neurocognitive Assessment, and Associated Risk Factors in Persons 50 Years and Older. Pathogens.

[34] Foroutan M., Vafae Eslahi A., Soltani S., Kamyari N., Moradi-Joo E., Magnaval J.-F., Badri M. (2024). Seroprevalence and Potential Risk Factors of Toxocariasis among General Population in Southwest Iran: Implications on the One Health Approach. J. Immunol. Res..

[35] Ardelean A.A., Lighezan R., Ursoniu S., Sprintar S.A., Oatis D.A., Mihu A.G., Lupu M.A., Olariu T.R. (2025). First Report on the Seroprevalence and Risk Factors Associated with *Toxocara* Infection in Blood Donors from Romania. Pathogens.

[36] Fenoy S., Cuéllar C., Aguila C., Guillén J.L. (1992). Persistence of immune response in human toxocariasis as measured by ELISA. Int. J. Parasitol..

[37] Araújo A.C., Villela M.M., Sena-Lopes Â., Farias N.A.d.R., Faria L.M.J.d., Avila L.F.d.C., Berne M.E.A., Borsuk S. (2018). Seroprevalence of *Toxoplasma gondii* and *Toxocara canis* in a human rural population of Southern Rio Grande do Sul. Rev. Inst. Med. Trop. Sao Paulo.

[38] Pezeshkian F., Pouryousef A., Omidian M., Mikaeili F., Safarpour A.R., Shojaei-Zarghani S., Sarkari B. (2023). Seroprevalence of Toxocariasis and Its Associated Risk Factors among Adult Population in Kavar District, Fars Province, South of Iran: A Cross-Sectional Community-Based Seroepidemiological Survey. Interdiscip. Perspect. Infect. Dis..

[39] Gee N.R., Mueller M.K., Curl A.L. (2017). Human-Animal Interaction and Older Adults: An Overview. Front. Psychol..

[40] Rubinsky-Elefant G., Hirata C.E., Yamamoto J.H., Ferreira M.U. (2010). Human toxocariasis: Diagnosis, worldwide seroprevalences and clinical expression of the systemic and ocular forms. Ann. Trop. Med. Parasitol..

[41] Despommier D. (2003). Toxocariasis: Clinical aspects, epidemiology, medical ecology, and molecular aspects. Clin. Microbiol. Rev..

[42] Ferreira I.B., de Souza Filho R.T., Lescano S.A.Z., Giuffrida R., Rodrigues D., de Faria Resende S.T., Figueiredo F.B., Kmetiuk L.B., Dos Santos A.P., Biondo A.W. (2025). High toxocariasis seroprevalence in a tri-border indigenous community (Brazil, Paraguay and Argentina): A One Health perspective. One Health.

[43] Santarém V.A., Pinto G.L.B., de Souza Filho R.T., Ferreira I.B., Lescano S.A.Z., Gonzáles W.H.R., Kosloski J., Ribeiro J., Giuffrida R., Dos Santos A.P. (2023). Risk factors for toxocariasis during incarceration: The One Health intervention approach. Sci. Rep..

[44] Lima V.d.S.V., Ferreira I.B.B., Rapchan G.G.d.S., Lescano S.Z., Rodrigues e Fonseca G., Giuffrida R., Kmetiuk L.B., Biondo A.W.W., Santarém V.A.A. (2025). Toxocariasis in children: Seroprevalence after 15 years in a major city in Brazil. Front. Pediatr..

