# Supplementary material for: Recycling Workers: Food Insecurity as a Novel Associated Risk Factor for Toxocariasis
Source: Pathogens. 2026 Sep 6;15(9):945. doi: 10.3390/pathogens15090945 (PMC13609950; doi:10.3390/pathogens15090945)
Supplement: Supplementary file 1 [file pathogens-15-00945-s001.zip › pathogens-4471040-supplementary.pdf]

Supplementary Table S1. Seropositivity and associated risk factors between anti-IgG against *Toxocara* spp. in non-recycling workers in southern Brazil.

	**Positive n (%)**	**Negative n (%)**	**OR (95% CI)**	***p****
	38 (38.0)	62 (62.0)		
**Gender**				0.506
Female	27 (71.1)	49 (79.0)	1 [Reference]	
Male	11 (28.9)	13 (21.0)	1.53 (0.59-3.93)	
**Age (interquartile)**				0.467
17-29 anos	10 (26.3)	15 (25.0)	1 [Reference]	
30-38 anos	8 (21.1)	18 (30.0)	0.67 (0.20-2.17)	
39-49 anos	9 (23.7)	17 (28.3)	0.80 (0.25-2.54)	
50-75 anos	11 (28.9)	10 (16.7)	1.63 (0.50-5.47)	
**Ethnicity**				1
Non-white	20 (52.6)	33 (54.1)	1 [Reference]	
White	18 (47.4)	28 (45.9)	1.06 (0.47-2.41)	
**Food_Insecurity**				0.792
No	19 (50.0)	34 (54.8)	1 [Reference]	
Yes	19 (50.0)	28 (45.2)	1.21 (0.54-2.75)	
**Handwashing_before_meals**				0.671
No	3 (7.89)	3 (4.84)	1 [Reference]	
Yes	35 (92.1)	59 (95.2)	0.60 (0.10-3.64)	
**Raw meat_intake**				0.478
No	21 (55.3)	40 (64.5)	1 [Reference]	
Yes	17 (44.7)	22 (35.5)	1.47 (0.64-3.38)	
**Raising_pets**				0.672
Cat	2 (5.26)	1 (1.69)	1 [Reference]	
Dog	24 (63.2)	40 (67.8)	0.32 (0.01-4.20)	
Dog and cat	12 (31.6)	18 (30.5)	0.36 (0.01-4.96)	
**Rinsing_vegetable_before_meal**				0.484
Chemical product	16 (44.4)	20 (33.3)	1 [Reference]	
Running water	17 (47.2)	31 (51.7)	0.69 (0.28-1.68)	
Soap	3 (8.33)	9 (15.0)	0.43 (0.08-1.79)	
Familiar income (minimum wage)				1
< 2500	2 (6.67)	3 (6.52)	1 [Reference]	
More than 2500	28 (93.3)	43 (93.5)	0.96 (0.14-8.66)	
